# Benefits of gene flow are mediated by individual variability in self‐compatibility in small isolated populations of an endemic plant species

**DOI:** 10.1111/eva.12437

**Published:** 2016-12-26

**Authors:** Christopher T. Frye, Maile C. Neel

**Affiliations:** ^1^ Natural Heritage ProgramMaryland Department of Natural ResourcesWildlife and Heritage ServiceWye MillsMDUSA; ^2^ Department of Plant Science and Landscape ArchitectureUniversity of MarylandCollege ParkMDUSA; ^3^ Department of Plant Science and Landscape Architecture and Department of EntomologyUniversity of MarylandCollege ParkMDUSA

**Keywords:** endemic, gene flow, index of self‐incompatibility, mating system, pseudo‐self‐fertility, *Trifolium virginicum*

## Abstract

Many rare and endemic species experience increased rates of self‐fertilization and mating among close relatives as a consequence of existing in small populations within isolated habitat patches. Variability in self‐compatibility among individuals within populations may reflect adaptation to local demography and genetic architecture, inbreeding, or drift. We use experimental hand‐pollinations under natural field conditions to assess the effects of gene flow in 21 populations of the central Appalachian endemic *Trifolium virginicum* that varied in population size and degree of isolation. We quantified the effects of distance from pollen source on pollination success and fruit set. Rates of self‐compatibility varied dramatically among maternal plants, ranging from 0% to 100%. This variation was unrelated to population size or degree of isolation. Nearly continuous variation in the success of selfing and near‐cross‐matings via hand pollination suggests that *T. virginicum* expresses pseudo‐self‐fertility, whereby plants carrying the same S‐allele mate successfully by altering the self‐incompatibility reaction. However, outcrossing among populations produced significantly higher fruit set than within populations, an indication of drift load. These results are consistent with strong selection acting to break down self‐incompatibility in these small populations and/or early‐acting inbreeding depression expressed upon selfing.

## 1 Introduction

Plant mating systems mediate the frequencies of outcrossing and selfing, which, in turn, strongly affect the amount and distribution of genetic variation within and among populations (Charlesworth, 2006; Duminil, Hardy, & Petit, 2009; Loveless & Hamrick, 1984; Young, Broadhurst, & Thrall, 2012). Population size and connectivity also affect amounts and patterns of genetic variation in that small, isolated populations have lower levels of standing genetic variation and increased inbreeding (Eckert et al., 2010; Heschel & Paige, 1995; Holsinger & Vitt, 1997; Jacquemyn, De Meester, Jongejans, & Honnay, 2012; Soulé, 1987; Young & Pickup, 2010). Specific effects of population size on inbreeding and fitness may have complex dependencies on life history characteristics of species (Angeloni, Ouborg, & Leimu, 2011), but inbreeding generally negatively affects fitness (Frankham, 2015).

Species that are of conservation concern due to recent reduction in population size through habitat loss may be at higher risk of fitness declines than chronically rare species (Holsinger & Vitt, 1997; Honnay & Jacquemyn, 2007). If population reduction is accompanied by increased isolation that eliminates gene flow among previously connected populations, increased selfing and mating among close relatives can reveal substantial genetic load that was masked in larger populations (Keller & Waller, 2002). The consequences of more frequent inbreeding within populations may be indicated by low fruit set or low‐quality seed (early‐acting inbreeding depression), as well as poor survival or reproduction of inbred progeny (late‐acting inbreeding depression).

At the same time, selfing can provide reproductive assurance under conditions of low pollinator density, low mate availability, or otherwise marginal environmental conditions (Jacquemyn et al., 2012; Kalisz, Vogler, & Hanley, 2004; Karron et al., 2012; Lloyd, 1992). Benefits of reproductive assurance in small and isolated populations appear to outweigh the benefits of cross‐pollination that are found in large populations (Delmas, Cheptou, Escarvage, & Pornon, 2014; Herlihy & Eckert, 2002; Holsinger, 2000; Igić, Bohs, & Kohn, 2006; Kalisz et al., 2004; Porcher & Lande, 2005). Shifts in the relative proportions of selfing and outcrossing within populations are the result of mating system evolution and the diversity of mating systems in plants is an indicator of the flexibility in responding to selection (Levin, 2012). Evidence is increasing that the mating system itself may respond adaptively to small population size and fragmentation through breakdown of self‐incompatibility (Busch, Joly, & Schoen, 2010; Karron et al., 2012; Stephenson, Good, & Vogler, 2000; Willi, 2009). Levin (1996) suggested this breakdown is often due to the action of modifier genes that alter the effectiveness of self‐incompatibility alleles (i.e., pseudo‐self‐fertility, PSF), a critical step in the evolution of self‐fertility.

Gene flow among populations can alleviate inbreeding effects by introducing variation from relatively unrelated individuals that masks genetic load and restores compatible mating types (Cheptou & Donohue, 2011; Frankham, 2015; Spielman, Brook, & Frankham, 2004; Young & Pickup, 2010). The interaction between a species' dispersal ability and the distribution of habitat in a landscape determines patterns of gene flow under natural conditions. Long‐term patterns of gene flow may be very different than current gene flow if habitat patches are smaller (fewer potential migrants) and are further apart (requiring longer dispersal distances) than they were under historical habitat distributions (Honnay & Jacquemyn, 2007).

Because mating system and gene flow are key to assessing the risks associated with small population size, the effects of crossing distance between individuals within and among populations have long been of interest to evolutionary biologists and conservation biologists (Edmands, 2007; Fenster & Sork, 1988; Frankham et al., 2011; Marsden, Engelhardt, & Neel, 2013; Weeks et al., 2011; Whitlock et al., 2013). Field studies linking the reproductive biology and mating system of species with an ecologically relevant scale of crossing distance can assist land managers in making informed decisions regarding the potential benefits associated with increased gene flow when conducting restoration activities or developing management plans (Marsden et al., 2013; Whitlock et al., 2013).

**Figure 1:**
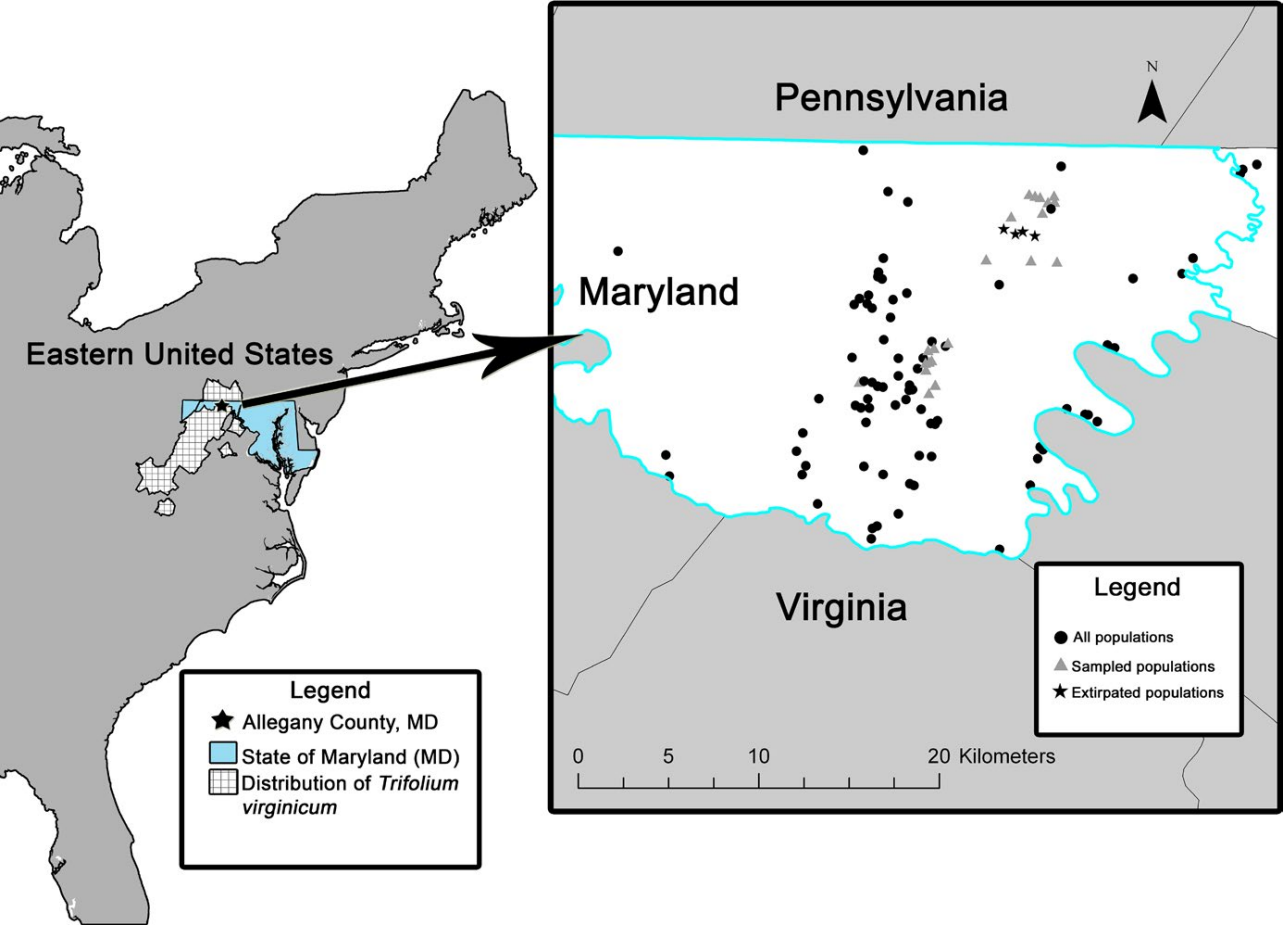
Geographic distribution of *Trifolium virginicum* in the eastern United States (hatched), location of the State of Maryland (blue), and the study area in Allegany County, Maryland (star). Inset: detail of the study area including sampled populations (triangles), unsampled but extant populations (filled circles), and extirpated populations (stars)

Thus, we sought to understand the effects of an artificial increase in gene flow in a species that exists in small and isolated populations, Kates Mountain Clover (*Trifolium virginicum* Small; Fabaceae), an endemic to the central Appalachian shale barrens. We investigated relationships between crossing distance and reproductive success across populations of different sizes and degrees of isolation. *Trifolium virginicum* is a perennial herbaceous plant species that is restricted to small habitat patches within the dominant woodland habitat on shale substrate. Shale barrens and *T. virginicum* are globally rare (NatureServe 2014). Within the shale barren region, however, *T. virginicum* has a relatively broad distribution, occurring in discrete barren patches within the Ridge and Valley Physiographic Province from southwestern Virginia and adjacent West Virginia, north through western Maryland and south‐central Pennsylvania (Figure 1).

As with many early successional habitats in eastern North America, shale barrens depend on periodic disturbance, such as wildfire, to retard succession to closed forest (Copeheaver, Fuhrman, Gellerstedt, & Gellerstedt, 2004; Foster et al., 2003; Norris & Sullivan, 2002; Tyndall, 2015). Lacking such disturbance, shale barren habitats have become restricted to small patches in which particularly harsh environmental conditions slow succession to woodland (Keener, 1983; Platt, 1951). Beyond forest succession, the shale barren region has experienced an increase in the number of potential barriers to gene flow via development and road construction (Copeheaver et al., 2004; Norris & Sullivan, 2002; Maryland Natural Heritage Program, Annapolis, MD). These changes in habitat structure leave *T. virginicum* in occupied areas within barrens that often cover only a few square meters. In Maryland, *T. virginicum* occurs on 95 barrens (Maryland Natural Heritage Program, Annapolis, MD). Seventy‐six percent of these barrens have <50 plants and only 11% have ≥100 plants. The largest known population in Maryland had ~373 plants as of a 2016 census. Barrens are separated from their nearest neighboring barren by a minimum of 0.2 km to a maximum of 12.8 km (median = 1.0 km). Extirpation of four populations during construction of an interstate highway created a 300‐ to 400‐m‐wide gap that effectively divided a northern group of barrens from a southern group (Figure 1).

*Trifolium virginicum* individuals are long‐lived, perhaps surviving many decades; marked plants at two sites have survived over 17 years. Plants have a deep taproot and produce multiple 2‐ to 3‐cm‐diameter spherical flower heads on 4‐ to 15‐cm‐long peduncles that lie prostrate on the ground and elongate with age. Pollination is likely affected by one or more native bee species. Fruits are slender legumes (pods) containing 1–3 seeds. The perianth and pods are long‐persistent; the small seeds (~2.2 to 2.7 mm diameter) are released in late summer upon disintegration of the inflorescence and lack obvious means for long‐distance dispersal.

The genus *Trifolium* is known to possess gametophytic self‐incompatibility (GSI; Lawrence, 1996). GSI is a widespread genetic system that enables hermaphroditic plants to avoid self‐fertilization and mating with close relatives by rejection in the pistil of pollen carrying the same S‐allele. Species in the genus *Trifolium* are known to have a large number of S‐alleles (Casey et al., 2010; Lawrence, 1996).

Given the broad distribution of small, isolated barrens within the extensive forested matrix and lack of adaptation for long‐distance seed dispersal, they likely represent relicts of a once more continuous distribution. If they are relicts, the extant patches have highly reduced population sizes and habitat areas that are far more isolated than they were prior to the last 100–160 years (Tyndall, 2015). This isolation of *T. virginicum* populations creates high risk of increased inbreeding, loss of genetic diversity, and loss of compatible mating types (S‐alleles) via drift. These risks could be ameliorated by managing the habitat between barrens to establish larger openings and to enhance gene flow by linking currently isolated patches or by intentional supplementation of small populations that have experienced no increase in population size in >30 years. In this study, we seek to understand the risks and benefits of increasing gene flow in *T. virginicum* to inform management choices that range from maintaining the status quo (within population mating) to supplementing populations using pollen, plants, or seed from distant populations. We gain this understanding by performing experimental crosses using self‐pollen (selfing), pollen from within sites (near‐cross), and pollen from distant sites (far‐cross). We quantify the effect of these cross‐types on reproduction (fruit set and seed weight) as measures of fitness, and examine variability in self‐compatibility (from hand self‐pollinations) among maternal plants and sites across a range of population sizes and degrees of isolation. Because small plant populations are often pollen‐limited (Knight et al., 2005), we characterize fruit set in open‐pollinated plants under natural conditions and examine relationships with population size and degree of isolation. We calculate the index of self‐incompatibility (ISI) as a species average and examine variation between type of outcross pollen (near or far) and variability among individual maternal plants. Thus, we go beyond the traditional usage of ISI in which species mating systems are categorized as self‐compatible (ISI < 0.2), mixed‐mating (0.2 < ISI > 0.8), or self‐incompatible (0.8 < ISI) (see Raduski, Haney, & Igić, 2011). Additionally, we test for reproductive assurance in the absence of pollinators (autogamy). Our analyses focus on answering the following questions:

1. Are there relationships between fruit set in self‐ and in open‐pollinated flowers with population size, or metrics quantifying the degree of population isolation?
2. Does cross‐type influence fitness (fruit set) and how much variability is due to maternal plants and sites?

We expected to find evidence of GSI in *T. virginicum* and concomitantly we predicted that the success of self‐pollination would be extremely low. However, preliminary data from five *T. virginicum* plants at three sites indicated great variation among maternal plants in ability to form fruit from hand self‐pollinations. Thus, we sought to investigate this variability in selfing success with a larger sample. We predicted that success of self‐pollination would vary with population size or degree of isolation. Specifically, we predicted that selfing success would increase with decreasing population size and increasing isolation due to strong selection for breakdown of self‐incompatibility in putatively mate‐limited populations experiencing little gene flow. In contrast, we predicted that fruit set in open‐pollinated plants would increase with increasing population size and decreasing isolation due to higher probabilities of compatible mates, larger floral displays to pollinators, and shorter pollinator flight distances. Finally, we predicted that far‐cross success would be consistently greater due to higher probabilities of delivering a novel S‐allele as well as the effects of heterosis.

The possibility that *T. virginicum* may be pseudo‐self‐fertile (PSF, sensu Levin, 1996) became evident during our field studies. Genes conferring PSF have been demonstrated in the genus by Atwood (1942) and Townsend (1965, 1966, 1969). *Trifolium repens* and *Trifolium pratense* are known to exhibit breakdown in self‐incompatibility due to PSF loci whose function may vary over time (Riday & Krohn, 2010; Yamada, Fukuoka, & Wakamatsu, 1989) or under high temperatures (Townsend, 1965). If PSF is a natural component of the mating system, we expected some selfing success, but with great variation across maternal plants (Levin, 1996). Variation in selfing success in early versus late flowers is also expected but because this experiment was not designed to test for PSF, we did not test for temporal variation.

## 2 Methods

### 2.1 Site selection

We used graph theoretic analysis as implemented by the computer program Conefor 2.6 (Saura & Torné, 2009, 2012) to select sites that varied independently in size and connectivity. Because of the large amount of information that can be gained with few data inputs, graph theory is being increasingly applied to conservation problems (Calabrese & Fagan, 2004; Neel, Tumas, & Marsden, 2014; Pascual‐Hortal & Saura, 2006). Graphs provide a spatially explicit representation of landscapes based on the distances at which habitat patches (termed nodes) are connected to one another into networks. We used a layer of all known populations of *T. virginicum* in Maryland as nodes. We calculated pairwise geographic distance among patches in ArcMap 10.0 (ESRI 2011). Census size was used to represent habitat size and quality for each node. Census size of each population is based upon counts of individuals at peak flower when plants are most visible.

We selected two graph metrics (IIC_connector_ and BC(IIC)) that provide meaningful and interpretable measures of connectivity (Bodin & Saura, 2010). IIC_connector_ measures each patch's contribution to connectivity as a stepping stone through which other patches of the network are connected. BC(IIC) measures the degree to which a node sits among other nodes in a network based on the number of shortest paths for movement between all pairs of nodes that pass through that node (Baranyi, Saura, Podani, & Jordan, 2011; Bodin & Saura, 2010). We assess the importance of each node using forms of IIC_connector_ and BC(IIC) that are calculated as the change in values between the full network and a network from which each focal node has been sequentially removed (called *d*IIC_connector_ and *d*BC(IIC)). *d*IIC_connector_ and *d*BC(IIC) are independent measures of connectivity (Bodin & Saura, 2010). The former measures the importance of a node based on how much connectivity would be reduced if the patch was lost. The latter measures the importance of a patch based on its centrality in an existing landscape.

To assess connectivity at multiple scales, we calculated patch importance values for *d*IIC_connector_ and *d*BC(IIC) for interpatch distances from 150 m (the minimum distance between two patches) to 36,000 m in 100‐m increments (measured from approximate population centroids). We graphically assessed the behavior across this range of distances and noted the distance(s) at which local maximum values (thresholds) were achieved. At these distances, the network of patches is particularly susceptible to changes in connectivity. We noted three such distances: 500, 1,000, and 1,850 m that we examined further.

We examined the rank of node importance values of *d*IIC_connector_ and *d*BC(IIC) at the three distances for use. Sites were categorized as connected if both metrics were >0 at two of the three distances and isolated if both metrics were zero at two of the three distances. Sites were subdivided into small (<50 plants), medium (≥50 and <100 plants), and large (≥100 plants) populations based on censuses by the Maryland Natural Heritage Program from 1984 to 2015. Sites were then evaluated for accessibility (e.g., not on private property) and appropriateness for study (e.g., supported >3 plants).

Twenty‐one sites that covered the range of size and connectivity we sought were located in a 10 km × 15 km area that lies within the Green Ridge State Forest in Allegany County, Maryland (Figure 1). We updated the census for these sites in 2013 (Table 1). Sites occurred on the same geological formation and all experience the same general climate, thus limiting the possibility of local adaptation to different environments. The population size distribution of the study sites roughly mirrors the distribution of all *T. virginicum* populations in Maryland (Maryland Natural Heritage Program, Annapolis, MD): thirteen (62%) of the study sites are classified as small populations versus 76% of all sites, five (19%) are medium populations versus 13% of all sites, and three (14%) are large populations, versus 11% of the total (Table 1).

**Table 1:** Study sites with number of *Trifolium virginicum* censused in 2013, connectivity category (>0 = connected, 0 = isolated) using *d*IIC_connector_ and *d*BC(IIC) at two of three dispersal distances (500, 1,000, 1,850 m) and number of maternal plants receiving each of the three crossing treatments, tested for autogamous selfing, and open‐pollination

Site	Census size (2013)	Population size category	Connectivity category	Number of mothers receiving crossing treatments	Autogamy	Open
1	64	Medium	Isolated	5	5	5
8	131	Large	Isolated	7	3	7
9	67	Medium	Isolated	4	0	4
15	8	Small	Isolated	1	1	1
32	130	Large	Isolated	7	3	7
33	46	Small	Connected	1	1	1
34	65	Medium	Connected	4	3	4
35	9	Small	Connected	1	0	2
38	158	Large	Connected	5	0	5
43	23	Small	Isolated	2	1	2
44	23	Small	Isolated	3	6	3
45	30	Small	Connected	2	3	2
46	9	Small	Isolated	1	1	1
51	9	Small	Connected	1	1	1
54	12	Small	Connected	1	0	1
55	54	Medium	Connected	5	2	5
57	46	Small	Isolated	3	1	3
60	6	Small	Isolated	1	1	1
74	19	Small	Connected	2	0	3
99	50	Medium	Isolated	2	1	2
100	38	Small	Isolated	2	2	2
*N *=* *21	Σ = 960			*N *=* *60	*N *=* *35	*N *=* *62
				Σ maternal plants = 157		

### 2.2 Selection of maternal plants at sites

At each site, we selected maternal plants for pollination treatments (detailed in the next section) and deployed pollinator exclusion bags prior to flowering (Table 1). The choice of experimental plants was based on a combination of distance from other plants and accessibility. Even within larger patches of habitat, *T. virginicum* individuals are often clustered in small microsites. To increase chances of sampling less related individuals, we selected maternal plants at each site that were separated by at least 5 m as a general convention. We continued selection of maternal plants until all such isolated clusters of plants at a site were utilized. If all plants at a site were within 5 m, we chose only a single plant.

For each maternal plant treated at each site, we chose one additional maternal plant that received no treatment to represent open‐pollination (Table 1). Finally, we chose 35 additional plants at 16 sites to test for autogamy (Table 1). These plants received no additional manipulation beyond bagging.

### 2.3 Crossing design and procedures

On each experimental maternal plant, we established three treatments reflecting distance from pollen source. Implementing all treatments on the same plant controls for maternal genotype. The selfing treatment (S) used pollen from within the same inflorescence. The near‐cross treatment (NX) used pollen from 20 to 130 anthers collected from 2 to 13 donors within the site that were ≥5 m from the maternal plant. The far‐cross treatment (FX) used pollen from 20 to 130 anthers from 2 to 13 fathers from sites at least 1 km distant. We pooled pollen from multiple fathers to increase the probability of delivering at least one compatible S‐allele. Variation in number of donors resulted from differences in the number of plants in populations and the number of flowers with pollen available. In some of the smallest populations, additional plants other than the focal maternal plant were needed to serve as pollen donors for the near‐cross; these plants were by necessity sometimes within 5 m. Pollen for the NX treatment was collected while at the site and was applied within 30–60 min of collection. Pollen for the FX treatment was collected 1–4 hr prior to application. Anthers were mixed in a vial and kept on ice until they were applied to the mothers. In some cases, entire heads (with attached peduncle) were kept overnight, and anthers were harvested from freshly open flowers.

All crosses were performed under field conditions and sites were visited daily (weather permitting) between May 5 and May 15, 2013 to perform crosses. Pollination treatments were performed on flowers when the banner was expanded and reflexed exposing magenta‐colored nectar guides to pollinators. We had previously determined that the stigma was receptive in this period as assessed by testing for peroxidase activity using a 3% solution of hydrogen peroxide (Dafni, Kevan, & Husband, 2005). All flowers were emasculated using forceps. Anthers were applied directly to the stigma in the self‐treatment and outcross treatments used stamens with dehiscing anthers from the NX or FX anther pools as appropriate. One to two anthers were haphazardly selected from the appropriate tube for application to the stigma using forceps. Forceps were dipped in ethanol and flamed before moving to the next treatment.

We attempted to complete all treatments on one maternal plant (ranging from 35 to 60 min per plant) on a single day. If few flowers were receptive during a single visit, we returned the following day in an attempt to pollinate at least five flowers per head per treatment. We succeeded in pollinating 897 flowers with an average of 5.5 flowers per treatment. The number of flowers per head in each pollination treatment varied because the number of flowers available for pollination during any time interval was unpredictable. In total, 180 heads on 60 individual maternal plants received manipulative treatments (S, NX, FX).

Flower heads from all manipulative treatments, flower heads testing for autogamy and heads from open‐pollinated plants were collected and brought to the laboratory for dissection after 4–6 weeks in the field. There was occasional loss of individual treatments on some maternal plants due to destruction of pollinator exclusion bags by wildlife. Percent fruit set $\left(\frac{\mathrm{number}\;\mathrm{of}\;\mathrm{flowers}\;\mathrm{producing}\;\mathrm{fruit}}{\mathrm{number}\;\mathrm{of}\;\mathrm{flowers}\;\mathrm{treated}}\times 100\right)$ was calculated for each treatment on each maternal plant. We determined seed weight (nearest 0.01 mg) from experimental crosses and open‐pollination using a balance.

### 2.4 Fruit set and number of seeds per pod in open‐pollinated flowers under natural conditions

We quantified natural fruit set from 62 open‐pollinated heads from 62 maternal plants at 21 sites. We also quantified the average number of flowers per head and the number of seed per pod in 1,199 legumes containing at least one seed. All statistics reported as mean ± standard error (SE) or median and range of values.

### 2.5 Data analyses

We performed all statistical analyses (with the exception of a generalized linear mixed model detailed below) using Systat 13 (Systat Software, Inc., San Jose, CA, USA). We calculated mean (±SE) seed weight for each crossing treatment in each site (S, 17 sites, *N* = 167; NX, 19 sites, *N* = 157; FX, 16 sites, *N *=* *150) and for open‐pollination (O, 17 sites, *N *=* *1,001). We tested for differences in seed weight among crossing treatments using a general linear mixed model with cross‐type as fixed effect and site as a random effect. We assessed variability in mating system using percent fruit set in each of the three manipulative pollination treatments remaining on maternal plants (S, NX, FX; *N *=* *55, 54, 50, respectively), open‐pollinated flowers (O, *N *=* *62), and heads testing for autogamy (A, *N *=* *35). Percent fruit set violated assumptions of normality (Shapiro–Wilk tests, *p *<* *0.05) so we report both mean and median values. We tested the significance of differences in the variances for manipulative treatments and open‐pollination using Levene's test, confirming that our data also violated assumptions of homogeneity of variances (*F* = 3.314_2, 135_, *p *=* *0.026).

We calculated the index of self‐incompatibility (ISI) following Raduski et al. (2011) as

ISI=1−(selfedsuccess/outcrossedsuccess)

We computed Spearman rank correlations between percent fruit set in self‐ and open‐pollination and population size at each site (*N *=* *21) using 1,000 bootstraps to assess the significance of the correlation. We also tested for relationship between self‐ and open‐pollination averaged within sites using Spearman rank.

We explored relationships between the calculated values for connectivity metrics (*d*IIC_connector_, *d*BC(IIC)) at 500, 1,000, and 1,850 m, and percent fruit set in self‐ and open‐pollination at each site (*N *=* *21) using Spearman rank correlation analyses.

We examined the effect of cross‐type on probabilities of fruit set using a hierarchical generalized linear mixed model (GLMM; as implemented by PROC GLIMMIX, SAS v. 9.4). GLMMs are the appropriate tool for analyzing non‐normal data with random effects (Bolker et al., 2008). We modeled fruit set as a binary outcome (failure = 0, success = 1) for each cross‐type (self, near, far) resulting from individual hand‐pollinated flowers (*N *=* *766), nested within maternal plants (*N *=* *46), which were nested within site (*N *=* *15). We restricted our analysis to the 46 maternal plants with no missing data (all cross‐types present) to control for maternal genotype. We specified a binomial distribution and a logarithmic link (logit) to transform the dichotomous outcome into a continuous variable (the log‐odds). The logit transformation allows us to establish a linear relationship between our binary outcome variable (fruit set) and the predictor variables (cross‐type as fixed effect and maternal plant and site as random effects). The log‐odds of fruit set and errors for cross‐type, maternal plant and site was estimated using residual pseudo‐likelihood (modeled in PROC GLIMMIX as the probability of fruit set failure). To account for the hierarchical nature of the data, log‐odds is calculated using different intercepts for random subjects (site, maternal plant nested within site) to estimate variance parameters of subject and subject × cross‐type interactions. The intercepts are the average log‐odds of fruit set for the near‐cross because this represents the most probable outcross event among maternal plants within sites. We estimate log‐odds solutions for the fixed effects (cross‐type = self, near, and far) predicting the probabilities of fruit set failure and test for significant differences in the probability of fruit set failure between cross‐types.

For each random subject and subject × cross‐type interaction, we report the variance parameter estimates, standard errors, and the percent of the total variance attributable to subjects by dividing the estimate by the sum of variances. We did not calculate log‐likelihood ratio tests for significance of random effects because these methods have not been resolved for binary data (Bolker et al., 2008).

Because log‐odds range from zero to positive infinity, we converted the log‐odds of fruit set to predicted probabilities (expressed as a percent) of fruit set success and failure for fixed effects using the equation

$$\phi_{ij}=\frac{e^{\eta ij}}{1+e^{\eta ij}}$$

where e takes a value of approximately 2.72, *ηij* is the log‐odds of failure, and *φ* is the probability of failure.

## 3 Results

The sample of 62 open‐pollinated *T. virginicum* heads yielded a total of 2,410 flowers with a mean of 38.3 (±8.7) flowers per head (median = 37, range = 22–61). Median fruit set per head was 48.7% (Table 2). Flowers matured centripetally within flower heads and each one was available to pollinators for a single day. These open‐pollinated heads yielded 1,199 well‐formed pods that each most often contained a single seed (mean = 1.06 ± 0.02, median = 1, range 1–1.81).

**Table 2:** Summary statistics for percent fruit set within heads in each treatment. Columns present statistics for distribution of the values, and rows represent treatments (S, self; NX, near‐cross; FX, far‐cross; A, autogamy; O, open‐pollination). Variances with the same superscript letter are not significantly different (*p *<* *0.05)

Cross‐type	Number of heads	Mean fruit set	Median fruit set	Range	Variance
S	55	49.7	50.0	0, 100	979.35^a^
NX	54	55.8	60.0	0, 100	1174.04^a^
FX	50	69.6	63.5	17, 100	581.62^b^
A	35	0.7	0	0, 9.8	N/A
O	62	49.3	48.7	0, 87	542.42^b^

**Figure 2:**
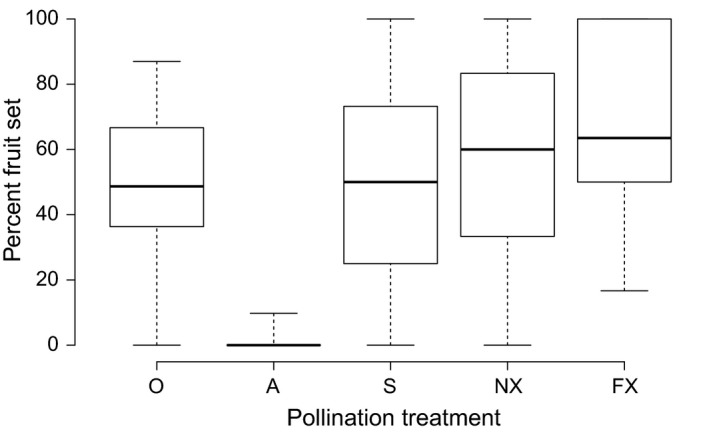
Percent fruit set within treatments for all maternal plants: Open‐pollination (O), bagged heads testing for autogamy (A), self‐pollination (S), near‐cross (NX), far‐cross (FX). Median marked by the central line within the box. The area of the box limits 25th and 75th percentiles (the interquartile range). Vertical lines extending from the box extend to minimum and maximum values

*Trifolium virginicum* set fruit upon selfing (median = 50%) and outcrossing (FX median = 63.5%; NX median = 60.0%) (Table 2; Figure 2). There was essentially no autonomous selfing based on fruit set in the 35 heads testing for autogamy (median = 0) (Table 2; Figure 2). There was no main effect or interaction of cross‐type and site on seed weight (*F*_3, 45_ = 1.08, *p *=* *0.22) so seed weight was eliminated as a variable of interest.

**Figure 3:**
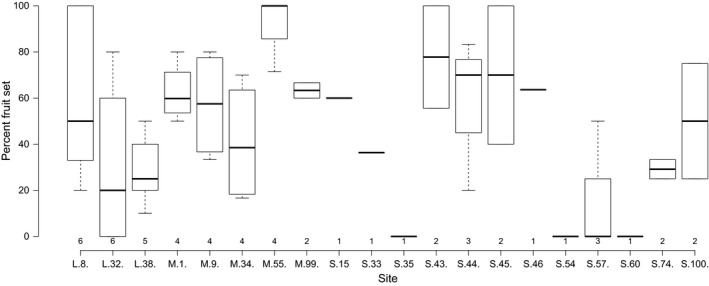
Variability in selfing success (percent fruit set) of maternal plants within 20 sites. On the horizontal axis, site census size (L, M, S) followed by site number is shown below the axis and sample size above the axis (no data for site 51). Median marked by the central line within the box. The area of the box (when *N *>* *1) limits 25th and 75th percentiles (the interquartile range). Vertical lines extending from the box extend to minimum and maximum values

The mean ISI value of 0.05 (*N *=* *53) would place *T. virginicum* in the category of self‐compatible species according to a survey of ISI values for >1,200 angiosperm taxa (Raduski et al., 2011). Observed values of ISI across maternal plants ranged from 1 to −4. This variation is obscured if only species‐level averages are reported (as in Raduski et al., 2011; Schoen & Lloyd, 1992), and if negative ISI values are set to zero (as in Raduski et al., 2011). These negative values indicate when selfing outperforms outcrossed matings. The success of self‐pollination varied dramatically among maternal plants finding instances of both full self‐compatibility (100% fruit set) and apparent self‐incompatibility (0% fruit set) (Table 2; Figure 3).

The mean ISI value indicated little difference in the overall success of self‐ versus outcross pollen in terms of fruit set when FX and NX, as outcross pollen, were combined. However, this result masks the contrasting ISI values for the two types of outcross. For the S/NX ratio, ISI of −0.12 (*N *=* *43) indicates higher fruit set on average with self‐pollen. By contrast, for the S/FX ratio, ISI of 0.21 (*N *=* *48) suggested mixed‐mating and a strong advantage to far‐cross pollen (see Appendix S1).

We found no significant correlations between population size and percent fruit set resulting from self‐ or open‐pollination. Self‐ and open‐pollination success within sites was positively but not significantly correlated (*R* = 0.32, *p *=* *0.16). As expected, open‐pollination success was positively correlated with population size (*R* = 0.32), but we found no clear evidence of pollen limitation as low fruit set was observed in some small, isolated populations (e.g., site 60, 0%; site 46, 9%) but not in others (e.g., site 15, 62%; site 100, 58%). Self‐success shows little relationship with population size (*R* = −0.02). We also found no significant correlations between fruit set in self‐ and open‐pollination with connectivity with neither the contribution of habitat patches as a stepping stone (*d*IIC_connector_) nor contribution of patch centrality (*d*BC(IIC)) at any of the three threshold distances (*N *=* *21 sites, maximum *R* = 0.22, *p *=* *0.34 between open‐pollination success and *d*IIC_connector_ at 1,850 m).

The GLMM revealed a significant effect of cross‐type while controlling for maternal plant and site characteristics (*F *= 4.66_2, 28_, Pr* > F *=* *0.02). The probability of fruit set failure for self‐ and near‐cross flowers was >0.55, whereas the probability of fruit set failure with far‐cross flowers is significantly reduced (<0.35) (Table 3). The probability of fruit set varied considerably across maternal plants within sites, but not among sites, demonstrating individual variability in mating system among maternal plants at all sites (Table 4).

**Table 3:** Log‐odds estimates and standard errors from a generalized linear mixed‐model (GLMM) analysis predicting the probability of fruit set for fixed effects (cross‐type). Significant (*p *<* *0.05) contrasts with near‐cross (intercept) are indicated by asterisks. Log‐odds estimates were converted to predicted probabilities of fruit set failure (PP_0_) in the last column. Probability of fruit set success (PP_1_) = 1 − PP_0_

Effect	Cross‐type	Estimate	Error	DF	*t* value	Pr > *t*	PP_0_
Intercept		−0.2065	0.2371	14	−0.87	0.40	55.2
Cross‐type	Self	0.2258	0.2909	28	0.78	0.44	55.6
Cross‐type	Far	−0.6480	0.3002	28	−2.16	0.04*	34.3
Cross‐type	Near	0					

**Table 4:** Logistic covariance parameter estimates, standard errors, and percent variance accounted for by random subjects and subject × cross‐type interactions. Variance in the log‐odds of fruit set modeled as a binary outcome (0 = failure, 1 = success)

Covariance parameter	Subject	Estimate	Error	Estimate/sum of variances
Intercept	Site	0.0300	0.1597	2.39%
Cross‐type	Site	0.1044	0.1686	8.21%
Intercept	Maternal plant(site)	0.4466	0.2621	35.13%
Cross‐type	Maternal plant(site)	0.6899	0.2684	54.27%

## 4 Discussion

We have shown that plants receiving pollen from another population (far‐cross) have significantly greater reproductive success than self‐ or near‐cross‐pollinations. The beneficial effects of gene flow are demonstrated by both a significantly higher probability of individual flowers setting fruit when treated with far‐cross pollen and positive ISI values for the S/FX ratio. At the same time, we found evidence of reproductive assurance resulting from successful hand self‐pollination in this putatively self‐incompatible plant. Most interestingly, we found great variation among selfed mothers that was independent of census population size and two measures of connectivity.

Census sizes of all sampled populations are small enough to generate population bottlenecks in S‐allele diversity (Thrall, Encinas‐Viso, Hoebee, & Young, 2014; Young et al., 2012) and to yield high inbreeding (Leimu, Mutikainen, Koricheva, & Fischer, 2006). The benefits of gene flow as mediated by artificial pollination could act by adding S‐allele diversity and by alleviating inbreeding depression (e.g., Frankham, 2015). The lack of relationship between self‐success and population size would appear to contradict evidence of the importance of population size in mediating the effects of inbreeding (Angeloni et al., 2011; Leimu et al., 2006). However, given that all of our populations have <150 individuals and most have <50 (Table 1), there is little room for variation in inbreeding effects. The lack of relationship of fruit set in self‐ and open‐pollination with connectivity measures could likewise indicate that gene flow does not vary over the distances that separate the remnant shale barrens. It is possible that the median separation of 1.0 km from the nearest neighbor is sufficient to functionally isolate barrens from one another and to preclude any gradient in gene flow. Additionally, variability in self‐compatibility in response to smaller population size and increase in isolation is expected as long‐lived species like *T. virginicum* may maintain levels of genetic variation that existed prior to the population declines for an extended period and may not exhibit expected effects of small, isolated populations. For example, Schleuning, Niggemann, Becker, and Matthies (2009) concluded that the longevity of *Trifolium montanum* has likely delayed extirpation of some populations. A lag in fitness declines in perennial species may be particularly long if there is strong selection against selfed progeny (Davies et al., 2015; Delmas et al., 2014; Young, Boyle, & Brown, 1996). Strong selection against inbred progeny in *T. virginicum* is suspected as census size estimates for some populations in 1984 were exactly the same in 2013; however, we cannot eliminate other factors such as poor pollinator visitation rates or habitat degradation.

Although we did find evidence for reproductive assurance, selfing success was significantly more variable than far‐cross or open‐pollination, ranging from instances of full self‐compatibility to complete failure indicating apparent rejection of self‐pollen (Figure 3). Although error is a potential explanation, it is unlikely because the selfing treatment required little manipulation. This variability has two potential explanations: (1) random drift of alleles modifying the self‐incompatibility reaction, that is, pseudo‐self‐fertility and (2) early‐acting inbreeding depression due to expression of deleterious/lethal recessives. Variation in self‐incompatibility among maternal plants has been observed in a wide range of species (Good‐Avila, Mena‐Alí, & Stephenson, 2008) including naturally rare or endemic species (Schleuning et al., 2009 *Trifolium montanum*; Busch et al., 2010 *Leavenworthia alabamica*; Alonso & Garcia‐Sevilla, 2013, *Erodium cazorlanum*).

We confirmed one characteristic of PSF identified by Levin (1996): higher fruit production with outcross pollen than self‐pollen but a nearly continuous distribution of fruit set following self‐pollination (Figure 3). Additionally, two components of PSF identified by Levin (1996), the age of flowers and the temperature at the time of treatment, may represent gene by environment interactions that may have played a role in the observed variability. Although we completed three pollination treatments on a single plant within 1 hr on a single day, treatments were applied to different plants throughout a day and replicate treatments occurred over a 2‐week period. Thus, there is the possibility that time (flower age) and temperature contributed to the observed variability in self‐ and near‐cross success. These results strongly suggest the presence of alleles (with perhaps environmental triggers) that modify the self‐incompatibility reaction. Our finding that variability in the probability of fruit set was explained by individual variability in self‐compatibility among maternal plants across all sites supports this hypothesis (Table 4).

Alternatively, variability in the success of self‐ and near‐cross‐pollination could be due to early‐acting inbreeding depression because the former is the most extreme form of inbreeding and the latter likely due to mating by individuals that were already closely related. According to theory, maintenance of a self‐incompatible genetic system is linked with strong inbreeding depression (Gervais, Awad, Roze, Castric, & Billiard, 2014). A self‐compatible mutant that arises within a self‐incompatible population should spread rapidly as long as the costs of selfing do not exceed the 50% transmission advantage and the benefit of reproductive assurance. The severity of inbreeding depression will be affected by genetic relatedness among individuals and the efficacy of purging in these small populations, both primarily functions of population size (Young & Pickup, 2010). Purging of genetic load has been invoked to explain why small populations persist despite increased inbreeding (Garcia‐Dorado 2015; Husband and Schemske 1996; Winn et al. 2011). However, the effectiveness of purging is dependent upon the distribution of deleterious alleles and severity of their effects. Recessive mutations with major fitness effects are more easily purged in an inbreeding population, whereas mutations of small effect may be fixed by drift (Carr & Dudash, 2003; Gervais et al., 2014) or sheltered by linkage to loci under frequency dependent selection such as the self‐incompatibility locus (Glémin, Bataillon, Ronfort, Mignot, & Olivieri, 2001). The fitness consequences of increased inbreeding in our populations could theoretically be ameliorated by purging of genetic load and strong selection against inbred progeny (Crnokrak & Barrett, 2002; Fox, Scheibly, & Reed, 2010). However, the fraction of mutation load fixed by drift (drift load) is expressed even under random mating and may form a feedback loop where drift load lowers population size, which in turn enhances drift load (Carr & Dudash, 2003; Willi, Griffin, & Van Buskirk, 2013). A highlight of our study was finding a significant heterotic benefit of far‐cross relative to near‐cross, which is the best indicator of drift load in populations. Fitness declines caused by drift load that is fixed within small and isolated populations cannot recover without increased gene flow (Willi et al., 2013).

We cannot distinguish between PSF and inbreeding depression with our data and doing so will be important for this species (and many other endemics) because of the implications for conservation. If *T. virginicum* exhibits PSF, then this may be an evolutionary adaptive mechanism that allows persistence of small and isolated populations by ensuring some reproduction and thus the demographic integrity of these small populations. However, the benefits of reproductive assurance gained by breakdown in self‐incompatibility may occur at a cost of reduced fitness in resulting progenies, and lifetime inbreeding depression may be particularly acute for long‐lived perennial species (Alonso & Garcia‐Sevilla, 2013; Delmas et al., 2014; Morgan, Schoen, & Bataillon, 1997). Given the current landscape of isolated patches, increasing the ratio of self‐ relative to outcross progeny may have long‐term population‐level‐fitness consequences as genetic relatedness increases among remaining individuals and inbreeding reduces fruit and seed set.

Importantly, we found that reproductive assurance can occur in *T. virginicum* through pollinator‐mediated selfing as determined analogously by hand‐pollination in our study. However, we found no evidence that autogamous self‐pollination is a reliable mechanism for reproductive assurance in absence of pollinators (Table 2; Figure 2); thus, pollinator abundance and floral attractiveness to pollinators in small populations will impact population persistence (Levin, 2012).

Current shale barren restoration and management strategies of selective tree removal and prescribed fire may theoretically restore available habitat for *T. virginicum* (see Tyndall, 2015). Although there are no data on *T. virginicum* dispersal distances, Matter, Kettle, Ghazoul, Hahn, and Pluess (2013) found that most dispersal events for the congener *T. montanum* in calcareous grassland fragments were <1 m and the maximum distance was 324 m. Thus, there is a high probability that *T. virginicum* seeds are dispersed near the maternal plant or into adjacent nonhabitat forest. Potential for colonizing newly created habitat patches will be limited unless they are extremely close to existing patches. Limitation in the colonizing ability of plant species is recognized as a major obstacle to habitat‐based restoration (Donohue, Foster, & Motzkin, 2000). Even if historical disturbance regimes are reinstituted, many previously occupied patches may remain unoccupied and patches remain fragmented. An understanding of long‐term gene flow patterns in *T. virginicum* represents an unmet need but is critical to understanding historical connectivity among populations. If historical patterns of gene flow are precluded by the current levels of isolation, then restoration goals should focus on re‐establishing gene flow between sites rather than on only single‐site management.

A reasonable hypothesis is that the historical landscape configuration facilitated gene flow, at least among proximal populations (e.g., those barrens occurring along the same ridgeline) and that changes in the landscape configuration and alteration in natural processes have resulted in severe fragmentation. Fire exclusion likely plays a role in restricting populations of *T. virginicum* to open‐canopy sites over shallow soils. Prescribed burns are potential management treatments that can increase populations. For example, a prescribed burn at one site in 1999 resulted in apparent population growth of *T. virginicum* according to census performed at dates preburn (May 1984, *N *=* *74) and postburn (May 2013, *N *=* *131). The question for long‐term conservation of *T. virginicum* and other species restricted or endemic to shale barrens is whether management of a few focal sites having relatively large populations (e.g., >100) is enough to conserve the evolutionary potential of these species. This management focus presumes that the larger barrens capture both general levels of genetic diversity and sufficient S‐allele diversity. Given the small sizes of even the largest populations and habitat areas, this assumption may not be warranted. Additionally, the success of single‐site management is measured by population increase, but the plants are long‐lived and there may be a substantial lag time in discerning demographic trends, a characteristic of extinction debt (Hanski & Ovaskainen, 2002; Tilman, May, Lehman, & Nowak, 1994). Future work on *T. virginicum* focuses on assessing whether the heterosis observed after far‐cross extends to lifetime fitness (germination to reproduction) of F1 progeny. Additionally, we need to assess genetic relatedness of individuals within sites to determine the extent to which inbreeding versus lack of S‐allele diversity explains the differential success between near‐ and far‐cross in this study. Finally, and critical for management, we need to elucidate long‐term (historical) patterns of gene flow to develop appropriately scaled reserves.

## Data archiving statement

Locality and population metadata for rare species is confidential but is available upon legitimate request with submission of a data sharing agreement with the Maryland Natural Heritage Program, Annapolis, Maryland, USA. All other data are available in Supporting Information files (Appendix S1) or by request to the authors.
